# Repetitive, mild traumatic brain injury results in a progressive white matter pathology, cognitive deterioration, and a transient gut microbiota dysbiosis

**DOI:** 10.1038/s41598-020-65972-4

**Published:** 2020-06-02

**Authors:** Mariana Angoa-Pérez, Branislava Zagorac, John H. Anneken, Denise I. Briggs, Andrew D. Winters, Jonathan M. Greenberg, Madison Ahmad, Kevin R. Theis, Donald M. Kuhn

**Affiliations:** 1 Research and Development Service, John D. Dingell VA Medical Center, Detroit, MI USA; 2 Department of Psychiatry and Behavioral Neurosciences, Wayne State University School of Medicine, Detroit, MI USA; 3 Department of Biochemistry, Microbiology and Immunology, Wayne State University School of Medicine, Detroit, MI USA; 4 Perinatal Research Initiative in Maternal, Perinatal and Child Health, Wayne State University School of Medicine, Detroit, MI USA; 5 Present Address: Stanford Behavioral and Functional Neuroscience Laboratory, Department of Neurosurgery, Stanford University Medical School, Stanford, CA USA

**Keywords:** Microbiology, Neuroscience, Anatomy

## Abstract

Traumatic brain injury (TBI) is often accompanied by gastrointestinal and metabolic disruptions. These systemic manifestations suggest possible involvement of the gut microbiota in head injury outcomes. Although gut dysbiosis after single, severe TBI has been documented, the majority of head injuries are mild, such as those that occur in athletes and military personnel exposed to repetitive head impacts. Therefore, it is important to determine if repetitive, mild TBI (rmTBI) will also disrupt the gut microbiota. Male mice were exposed to mild head impacts daily for 20 days and assessed for cognitive behavior, neuropathology and disruptions in the gut microbiota at 0, 45 or 90 days after injury. Deficits in recognition memory were evident at the late post-injury points. Brains show an early increase in microglial activation at the 0-day time point that persisted until 90 days post-injury. This was compounded by substantial increases in astrocyte reactivity and phosphorylated tau at the 90-day time point. In contrast, changes in the microbial community were minor and transient, and very few differences were observed in mice exposed to rmTBI compared to controls. While the progressive emergence of white matter damage and cognitive alterations after rmTBI resembles the alterations observed in athletes and military personnel exposed to rmTBI, these changes could not be linked to systematic modifications in the gut microbiota.

## Introduction

Traumatic brain injury (TBI) results from a blow to the head and its severity can range along a continuum from mild (e.g., brief change in mental status or consciousness) to severe (e.g., extended unconsciousness, coma, prolonged amnesia) to fatal. TBI is one of the most common neurological diagnoses in the US^1^, with a CDC estimate of approximately 2.5 million people on an annual basis. About one-third of all injury deaths arise from TBI and roughly 90,000 persons experience long-term disability yearly as a result of brain injury^2^. The cost of lifetime care for survivors of TBI has been estimated at $0.6-$1 million per individual with a total annual cost to the United States of ~$60 billion^3^, reflecting the severity of the dysfunction that results from this injury.

Perhaps the form of TBI that has garnered the greatest scrutiny recently, in the public eye as well as within military, scientific and medical communities, is repetitive, mild TBI (rmTBI). Military operations in Iraq and Afghanistan are revealing that TBI accounts for about 28% of all combat casualties^4^ approximately 85% of which are mild^4–6^. More than 2.4 million US military personnel have served on multiple combat tours, and the US Defense and Veterans Brain Injury Center has estimated that ~413,000 military service members have been diagnosed with mTBI from 2001–2011 (https://dvbic.dcoe.mil/dod-worldwide-numbers-tbi). Besides the military, collision sports are another setting in which rmTBI occurs with high frequency. Approximately 214 million children and adults participate in various sports per year in the USA alone^7^ and sports-related head injuries account for about 20% of all TBIs^8^. The majority of these injuries are also classified as mild to moderate^9–11^. An overriding concern in rmTBI is the possibility that repeat injuries may synergize with previous ones and their effects become cumulative^12^, possibly as a result of glial priming that occurs with advancing age^13^. Regardless of the setting in which rmTBI occurs, it has been estimated that approximately 20% will develop chronic comorbidities^8^ that can include psychiatric disorders (e.g., PTSD, depression, substance abuse)^14^ and a wide range of other medical conditions (e.g., pain, insomnia and fatigue, neuroendocrine dysfunction)^15^.

A perplexing systemic manifestation of TBI is gastrointestinal dysfunction, which can include mucosal damage, increased intestinal permeability and histopathological alterations in intestinal villi^16–19^. The metabolic alterations that accompany more severe TBI often require enhanced nutritional support to counter the “resistance to renutrition” that is seen in this and numerous other hypercatabolic situations^20,21^. Taken together, these latter complications of TBI suggest involvement of the gut microbiota. The GI system of humans and most other mammals is inhabited by a very large number of bacteria, viruses, fungi and archaea. Collectively, these microorganisms make up the gut microbiota. It has been estimated that the gut contains 100 trillion cells and these cells express > 150-fold more unique genes than the human genome^22^. The commensal members of the gut microbiota support human health but disruption in it has been implicated in a large number of clinical and physiological disorders [see^23–25^ for reviews]. Emerging preclinical studies are associating alterations in the gut microbiota with TBI but the majority of these imparted single, very severe head injuries and used relatively short survival times post-impact^26–29^. In light of the fact that the majority of military and sports-related TBIs are repeated and mild in nature, and considering that the fecal microbiota is altered for more than 20 years in humans after TBI^30^, we have evaluated the gut microbiota response to rmTBI involving 20 mild head impacts and longer survival times post-injury. The results show a progressive emergence of white matter damage, cognitive deterioration and a mild, transient gut dysbiosis.

## Materials and Methods

### Animals

Male C57BL6/J mice (8 weeks of age) were purchased from Envigo (Indianapolis, I.N.) and housed individually in a room with constant temperature and humidity and with alternating 12 hr periods of light and darkness. The use of male mice is justified in light of the fact that the overwhelming majority of military personnel (>86%), Veterans (>90%), and athletes (e.g., football players, >99%) exposed to repetitive head impacts are male. All mice used in these studies were from the same cohort and assignment to treatment groups was random. Mice were given *ad libitum* access to water and standard rodent laboratory chow (LabDiet 5001). Stressors such as noise and handling by multiple persons were avoided and mice were monitored daily for signs of distress or injury until the experimental endpoint. The Institutional Care and Use Committee of Wayne State University approved the animal care and experimental procedures (IACUC 19-03-0993). All procedures were also in compliance with the NIH *Guide for the Care and Use of Laboratory Animals* and were conducted in compliance with ARRIVE guidelines.

### rmTBI

Mice were anesthetized with isoflurane and exposed to a total of 20 head impacts (1 per day for 5 days [Monday-Friday with weekends off]) using a 30 g weight dropped from 1 meter, using a modification of our previously published method^31–33^. Our improved method uses a saloon door–style platform that ensures minimal resistance to movement on head impact and results in impact-induced acceleration^32^. The mouse’s head was positioned on the path of the drop weight and the intact scalp was impacted in the middle line between bregma and lambda. Control mice were anesthetized in the same manner as treated mice but were not exposed to head impacts. The amount of time required for recovery of the righting reflex (ROR) in treated mice and controls was recorded after each head impact. Mice were sacrificed by decapitation at 0, 45, or 90 days after the last head impact. Groups are referred to hereafter as follows: controls- 0, 45 or 90 day (Con-0, Con-45, Con-90) and rmTBI- 0, 45 or 90 day (TBI-0, TBI-45, TBI-90). All groups contained 6 mice except the Con-90 group which contained 5 mice. After sacrifice, brains were removed and placed into buffered 4% paraformaldehyde for 2 days, placed into cryoprotectant (20% sucrose in 1X PBS) and stored at 4 °C until immunohistochemical processing. The caecum of each mouse was also dissected free from the intestinal tract and its contents were weighed and stored at −80 °C until processed for DNA isolation.

### Novel object recognition (NOR) test

This test is used to evaluate recognition memory and it is based on the innate tendency of rodents to explore their environment. This natural inclination for exploration allows for testing whether a mouse can discriminate between a familiar and a novel object. Mice were tested in this paradigm prior to sacrifice at 0, 45, and 90 days post-injury. The NOR test was performed according to previous studies^34^ with some modifications. Briefly, mice from each group (n = 5–6) were individually habituated to the experimental cage (polycarbonate group I with a filter top) without any bedding for a period of 5 min. In the acquisition phase (24 h after habituation), two identical objects (A and B, which consisted of 5 × 2.5 cm plastic bottle caps) were positioned oppositely to each other on the cage and about 3–4 cm away from the walls. Animals were allowed to explore both objects for a 10 min period. During the memory recognition assessment phase that was assessed 10 min thereafter, one of the objects (A or B, the one explored less at acquisition phase to avoid a possible and confounding exploration bias) was replaced by a novel one (C, which consisted of a 6 × 2 cm ceramic dish), and the mouse exploratory behavior was analyzed over a 10 min period. Exploration of an object was defined as rearing on the object, sniffing it at a distance of less than 1 cm and/or touching it with the nose. Successful recognition was represented by preferential exploration of the novel object over the familiar object. After each session, the objects and cages were scrubbed with a tissue soaked in 96% ethanol and paper towel-dried to ensure that no olfactory cues were present. The time spent by each mouse exploring each object was recorded by an observer blinded to the treatment. The percentage of time spent exploring each object (novel vs familiar) was plotted using the total time investigating to normalize measures among individuals.

### Immunohistopathological analyses

Our previous studies showed that large white matter regions such as the optic tract were targeted for damage when using a greater mass drop weight (95 g^32^) so the present studies focused on this area as well. Brains from 3–4 subjects from each group were sectioned in the coronal plane on a cryostat to a thickness of 50 µm within the Bregma coordinates of −1.455 mm and −1.755 mm. A bilateral section per animal was incubated with the following primary antibodies at 4 °C overnight as described in our previous work^31,32^: anti-glial fibrillary acidic protein (GFAP, 1:500; LabVision, Fremont, CA); anti-ionized calcium-binding adapter molecule 1 (Iba1, 1:500; Agilent Dako, Santa Clara, CA); or anti-phospho-tau (p-tau, AT8, 1:500; Thermo Scientific, Rockford, IL). Primary antibody amplification was achieved using the Vectastain Elite ABC kit (Vector Labs, Burlingame, CA). All commercially available antibodies were validated by the manufacturers. Sections were then washed with 1 mL of 1X phosphate-buffered saline (PBS)/0.1% Triton X-100 three times for 5 minutes each. After the last wash, sections were incubated in 1X PBS for 5 minutes. The PBS was removed, and 1 mL of diaminobenzidine staining solution (Vector Labs, Burlingame, CA) was added to each section and incubated for 5 to 10 minutes at room temperature until sufficient color developed. Diaminobenzidine staining solution was removed, and 1 mL of 1X PBS was added to each section to stop the diaminobenzidine reaction. Sections were mounted on Fisher SuperFrost Plus Slides and thoroughly dried. Sections were dehydrated through graded ethanol washes, incubated in Citrisolv for 5 minutes, and coverslipped with Permount. Slides were allowed to dry overnight before viewing. Images were acquired at X10 magnification using an Olympus BX51 fluorescence microscope with a DP71 camera. All sections stained with each antibody were processed and imaged simultaneously for comparison purposes. The area through the optic tract was scanned for relative pixel density as an indirect measure of staining intensity using ImageJ software version 1.48 v (NIH, Bethesda, MD; http://imagej.nih.gov/ij).

### 16S rRNA gene sequencing

DNA was extracted from caecum contents (~200 mg wet weight) using QIAamp PowerFecal DNA kits and sample DNA concentrations were determined using a Qubit 4 Fluorometer and ranged from 70–100 ng/µl. Samples (n = 5–6 mice per group) were sequenced in duplicate on an Illumina MiSeq system using a 2 × 250 cycle V2 kit following Illumina sequencing protocols and with Illumina reagents following the procedures detailed by Kozich and colleagues^35^. The 16S rRNA gene primers used targeted the V4 region of the gene (forward primer: 5′-GTGCCAGCMGCCGCGGTAA-3′; reverse primer: 5′-GGACTACHVGGGTWTCTAAT-3′). The raw 16S rRNA gene sequences from the paired fastq files were processed with the Divisive Amplicon Denoising Algorithm (DADA2), a model-based approach for correcting amplicon errors without constructing operational taxonomic units^36^. Amplicon reads were processed with the DADA2 pipeline (v 1.12.1) to obtain merged, denoised, chimera-free, inferred amplicon sequence variants (ASVs) suitable to identify fine-scale variation. ASVs were defined by 100% sequence similarity, and analyzed using DADA2 in R (v 3.6.2), according to the online MiSeq protocol (https://benjjneb.github.io/dada2/tutorial.html), with some modifications that included truncation lengths of 240 bp and 160 bp and a maximum number of expected errors of 2 bp for forward reads and 7 bp for reverse reads. Sequences were classified using the “silva_nr_v132_train_set” database after removal of sequences derived from Archaea, Chloroplast, or Eukaryota.

The bacterial community data were thereafter visualized and statistically analyzed using PAST software (v3.20^37^). Microbiome diversity was characterized in terms of α-diversity using the Chao1 (i.e. community richness) and Shannon and Simpson (1-D) (i.e. community heterogeneity) indices. β-diversity was assessed using the Jaccard (i.e. shared composition) and Bray-Curtis (i.e. shared structure) indices based on relative abundance data. High-dimensional class comparisons were carried out with linear discriminant analysis effect size (LEfSe) in an on-line interface^38^ using default parameters with the exception that LDA score was set to 3.0. Heat maps were generated using MetaboAnalyst 4.0^39^.

### Statistical analysis

ROR was analyzed using repeated-measures two-way ANOVA (treatment X time) followed by Bonferroni’s multiple comparison’s tests using GraphPad Prism (v6.07) for Windows (GraphPad Software, La Jolla, CA, USA, www.graphpad.com). NOR test results and immunohistochemical data were analyzed using two-way ANOVA followed by Bonferroni’s multiple comparison’s tests in Prism. The indices for α-diversity were obtained using PAST software (v3.20) and analyzed with two-way ANOVA in Prism. The indices for β-diversity were calculated and analyzed using PAST. β-diversity results were analyzed using a two-way NPMANOVA, and *post hoc* comparisons were made with one-way NPMANOVAs. Taxonomic distributions at the phylum level (treatment X phylum) were analyzed with a two-way ANOVA followed by *post hoc* comparisons using Tukey’s test in Prism. Taxonomic classifications below phylum level were analyzed with a two-way ANOVA with Bonferroni *post hoc* tests. LEfSe analyses were carried out using the “all-against-all” parameter and an LDA score > 3.0.

## Results

### Neurological, behavioral and histopathological changes after rmTBI

#### rmTBI and recovery of righting reflex

**Figure 1:**
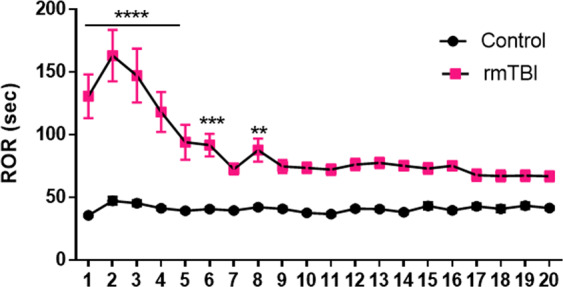
Effect of rmTBI on ROR. Mice were subjected to 20 head impacts and ROR was recorded after each impact daily. Controls were anesthetized but were not subjected to head impacts. The rmTBI group contained 18 mice and the control group contained 17 mice. The symbols indicate ****p < 0.0001; ***p < 0.001 and **p < 0.01.

The data in Fig. 1 show the time required for ROR over the course of treatment. Analyses with repeated-measures 2-way ANOVA revealed that the main effect of head impacts (F(19,824) = 6.55; p < 0.0001), treatment (F(1,824) = 312.3; p < 0.0001) and their interaction (F(19,824) = 5.63; p < 0.0001) were all significant. Two things are notable in Fig. 1. First, the initial increase in ROR during rmTBI was significant (p < 0.01-p < 0.0001; Bonferroni’s tests) but ROR recovered to control levels after 8 days. Second, for the last 12 days, the rmTBI group did not differ statistically from the control group with respect to ROR. Therefore, the head impacted mice could not be differentiated from controls by the end of treatment (i.e., do not appear injured). This result repeats earlier work published by our lab that used a 95 g weight vs the 30 g weight used presently^32^.

#### rmTBI results in a progressive emergence of cognitive impairments and white matter pathology

**Figure 2:**
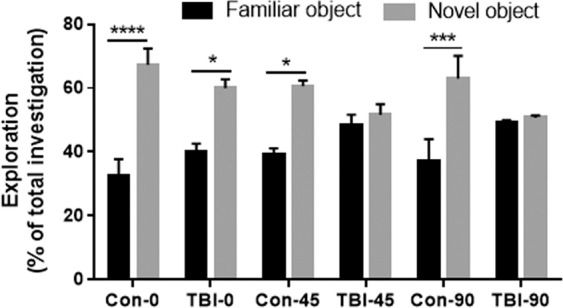
Effects of rmTBI on recognition memory (NOR test) at 0, 45, and 90 days post-injury. The percentage of time spent exploring each object (novel vs familiar) was plotted using the total time investigating to normalize measures among individuals. Mice were subjected to 20 head impacts and tested in the NOR paradigm prior to sacrifice at each time point. Each group contained 5–6 subjects and bars represent mean ± SEM values. The symbols indicate ****p < 0.0001; ***p < 0.001 and *p < 0.05.

Mice were analyzed for cognitive deterioration, a well-known behavioral symptom of chronic traumatic encephalopathy (CTE). Figure 2 shows that recognition memory was altered by rmTBI at the 45- and 90-day time points. There was a main effect of time (F(5,56) = 11.93, p < 0.0001) and treatment X time interaction (F(5,56) = 3.24, p < 0.05) but the main effects of treatment were not significant. Mice from both Con-0 (p < 0.0001) and TBI-0 (p < 0.5) groups discriminated the familiar object from the new one, indicating no effects of rmTBI on memory at this time point. While Con-45 mice discriminated between the familiar and novel object (p < 0.05), animals from the TBI-45 group did not. As it was observed at the 45-day time point, Con-90 discriminated between familiar and novel objects (p < 0.001), whereas mice in the TBI-90 group did not (Fig. 2).

Mice were analyzed for white matter pathology at each time point. We have previously shown that white matter tracts appear to be targeted when fewer impacts are administered using a higher weight^32^, so the following results focused on the optic tract.

**Figure 3:**
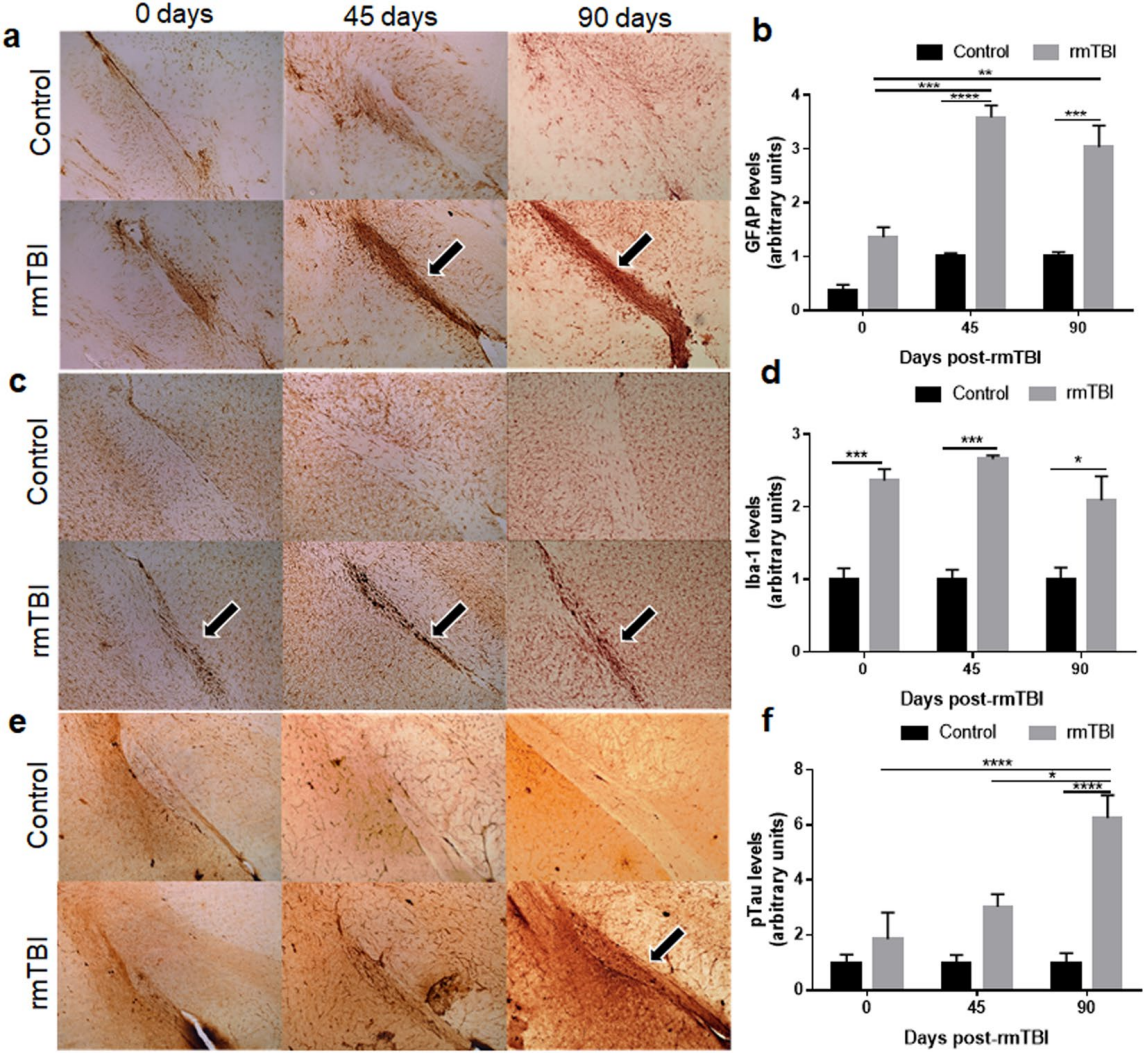
Effects of rmTBI on astrocyte (**a**), microglial (**c**) and p-tau (**e**) immunoreactivity in the optic tract. Levels of GFAP (**b**), Iba-1 (**d**) and p-tau (**f**) among the treatment groups quantified by pixel density from immunohistochemical analyses. Mice were subjected to 20 head impacts and brains were harvested at the indicated times and subjected to immunohistochemical analysis using antibodies against astrocytes (GFAP), microglia (Iba-1) or p-tau (AT8). Results are presented as means ± SEM. Groups are indicated as follows: Con-0 (control-0 days), TBI-0 (rmTBI-0 days), Con-45 (control-45 day time point), TBI-45 (rmTBI-45 day time point), Con-90 (control-90 day time point) and TBI-90 (rmTBI-90 day time point). The symbols indicate the levels of significance as follows for the indicated comparisons: ****p < 0.0001, ***p < 0.001, **p < 0.01 and *p < 0.05.

The effect of rmTBI on astrocyte reactivity (GFAP expression) is presented in Fig. 3a,b. There were statistically significant effects of treatment (F(1,15) = 96.11, p < 0.0001), time (F(2,15) = 21.07, p < 0.0001), and treatment X time interaction (F(2,15) = 5.88, p < 0.05). First, rmTBI does not have significant effects on astrocyte reactivity at the 0-day time point. Second, compared to time-matched controls, rmTBI caused a significant increase in GFAP at 45 days post-injury (p < 0.0001) that maintained until the 90-day point (p < 0.001; Fig. 3b and see black arrows in Fig. 3a). Third, astrocyte reactivity increased for the TBI-45 (p < 0.001) and TBI-90 (p < 0.01) groups compared to TBI-0. These results show that mice exposed to rmTBI have the same GFAP levels as non-impacted controls initially but signs of pathology emerge over time.

Sections were also stained for Iba1 to assess microglial reactivity in controls and rmTBI mice. The results show a slightly different pattern of response as seen for astrocytes (Fig. 3c,d). The main effect of treatment was statistically significant (F(1,15) = 78.98, p < 0.0001) but the effects of time or treatment X time interaction were not. rmTBI induced significant increases in microglial activation at all time points compared to their respective controls (p values ranging from 0.05 to 0.001; Fig. 3d and see black arrows in Fig. 3c). However, there were no differences between rmTBI groups, indicating that the microglial activation started shortly after injury and persisted through the latest time point.

Finally, sections from control and rmTBI mice were evaluated for the expression of p-tau, a hallmark sign of CTE neuropathology and the results are presented in Fig. 3e,f. The main effects of treatment (F(1,18) = 31.52, p < 0.0001), time (F(2,18) = 7.4, p < 0.01), and the treatment X time interaction (F(2,18) = 7.4, p < 0.01) on p-tau expression in the optic tract were significant. It can be seen that p-tau showed a more delayed increase with rmTBI than was shown for GFAP and Iba1, as only the TBI-90 group increased from its respective control (p < 0.0001; Fig. 3f and see black arrow in Fig. 3e). Levels of p-tau in both the TBI-0 (p < 0.0001) and the TBI-45 (p < 0.05) groups show a lower expression than the late rmTBI group at 90 days. These same neuropathological changes were seen in other white matter and gray matter areas in agreement with our previous studies^32^.

### Changes in the gut microbiota after rmTBI

#### Alterations in gut microbiota diversity

**Figure 4:**
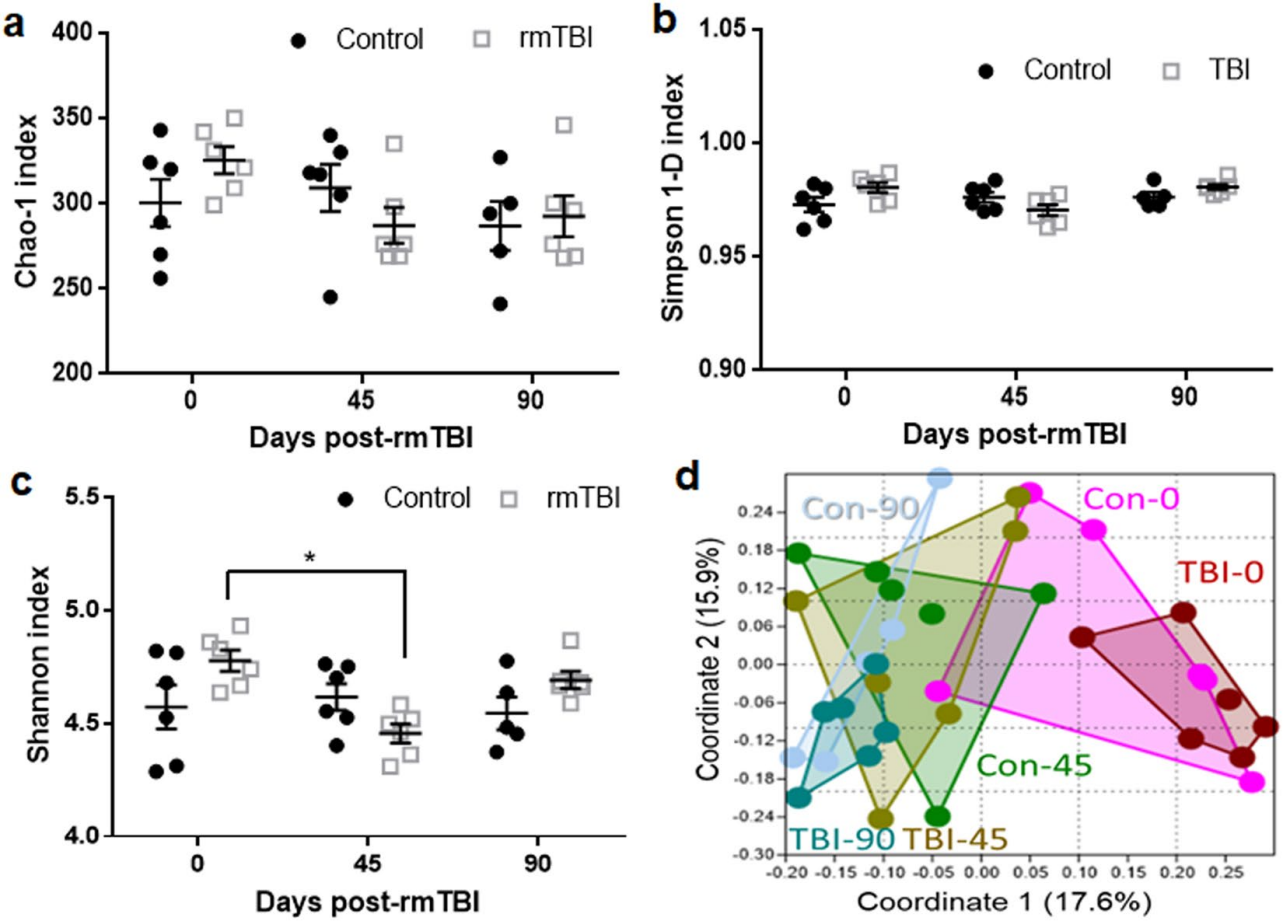
Effects of rmTBI on α- and β-diversity. Data for α-diversity are presented as the indices of Chao-1 (**a**), Simpson 1-D (**b**) and Shannon (**c**). Data are plotted as mean ± SEM. Each group contained 5–6 mice. The symbol * in panel C represents the significance level for the indicated *post hoc* comparisons of p < 0.05. Data for β-diversity (**d**) is presented as a PCoA and shows differences in the similarities of the gut microbiota clustering profiles for rmTBI and control groups for the 0 day, 45 day and 90 day time points using the Bray Curtis index for community structure.

The number of sequences was not altered by rmTBI at any time point and the values ± SEM for each group are as follows: Con-0: 44146 ± 1651; TBI-0: 47984 ± 2285; Con-45: 49492 ± 3585; TBI-45: 51753 ± 4405; Con-90: 47365 ± 2832; and TBI-90: 49770 ± 3778. Figure 4 presents an analysis of the effects of rmTBI on microbial α-diversity and β-diversity. Figure 4a shows results using the Chao-1 index as a measure of α-diversity. It can be seen that community richness was not significantly altered at any time point in either treatment group. Similar to the results of the Chao-1 index, α-diversity measured with the Simpson 1-D index revealed no significant effects of treatment, time, or these two factors interaction (Fig. 4b). However, while the interaction between treatment X time using the Shannon index was significant (F(2,29) = 5.1, p < 0.05), the main effects of treatment or time were not. These measures of community heterogeneity indicated that α-diversity was significantly reduced by rmTBI at the 45-day time point compared to the 0-day time point (Fig. 4c). Results from analysis of β-diversity using the Bray-Curtis index as a measure of community structure are presented in Fig. 4d. In general, the rmTBI and control groups clustered together for each time point (e.g., rmTBI and controls at the 0-day time point) and apart from the other time points (i.e. 45 or 90-day time points). Two-way NPMANOVA analyses revealed that the main effect of time was significant (F(1,29) = 1.09, p < 0.001) but neither the main effect of treatment nor the treatment X time interaction reached significance. Finally, when the taxonomic identities of the ASVs with > 0.5% relative abundance were arrayed in a heat map, the rmTBI:control group pairings showed very similar patterns of abundance for each time point and the most obvious differences occurred across time (i.e., from 0d to 45d to 90d) and not treatment. This heat map is included as Supplemental Fig. 1.

#### LEfSe analysis for biomarkers

**Figure 5:**
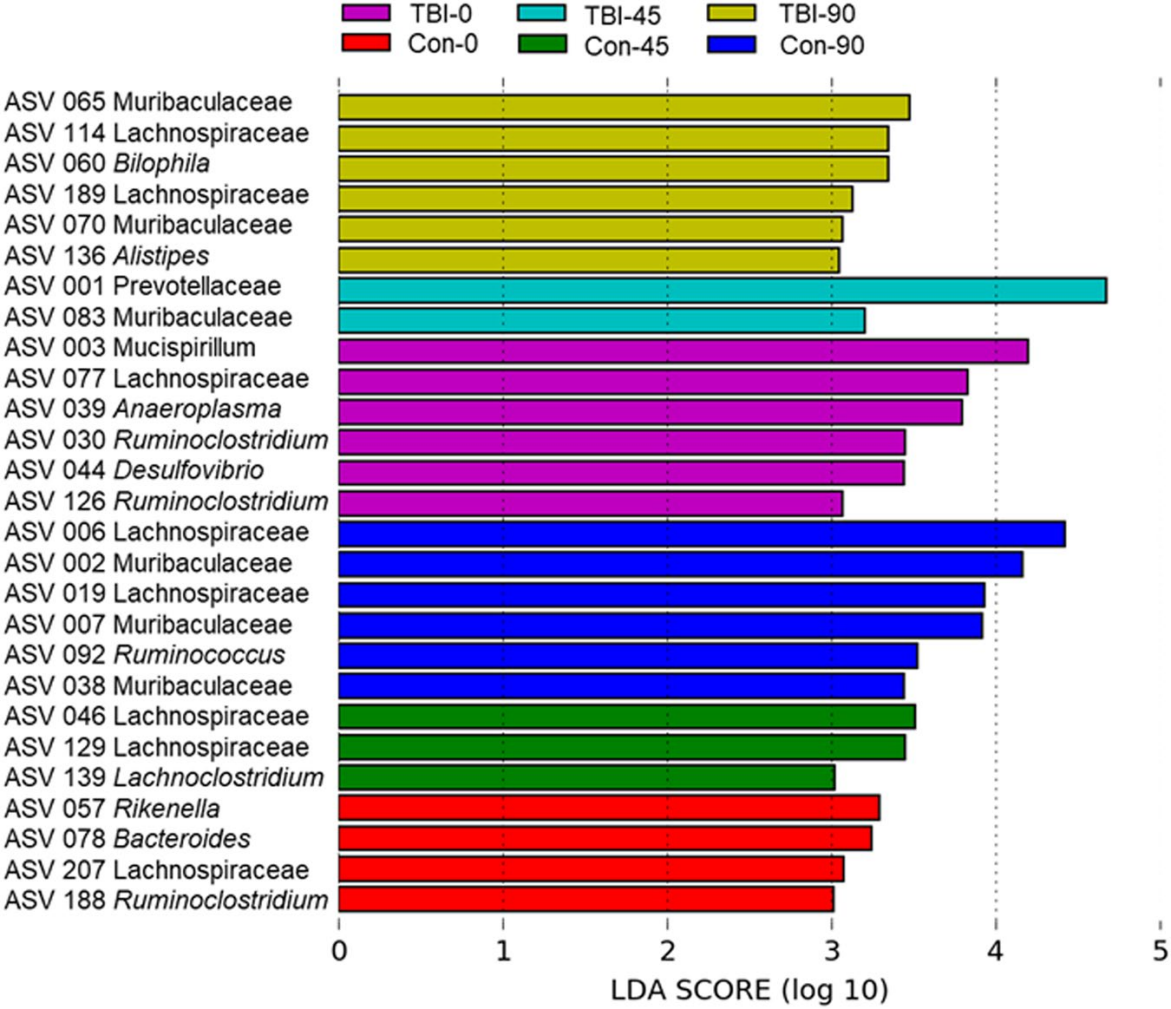
Bacterial taxa that were differentially abundant across treatments. LEfSe was carried out using the Galaxy Project and the results are displayed as bars the length of which is indicative the linear discriminant analysis score for each ASV. The taxonomic identity of each ASV is indicated to the left of each bar. All groups are statistically significant compared to each other (LDA > 3.0).

Segata and colleagues^38^ propose LEfSe as a means of biomarker discovery by finding taxa that consistently explain the differences between two or more types of microbial communities. The ASVs that characterize each treatment group, along with their taxonomic identities, are presented in Fig. 5. Clearly, the controls groups were represented by specific taxa that were different from the rmTBI groups and differences were also seen within each group with respect to time (e.g., TBI-0 vs TBI-45). Of the 27 ASVs that emerged from the LEfSe analysis, most could be identified to the level of family (N = 16) or genus (N = 11). In addition, the majority of these taxa are in the phyla Bacteroidetes (N = 10) and Firmicutes (N = 13), with two in Proteobacteria and one each in Deferribacteres and Tenericutes. The only group not dominated by biomarkers in Bacteriodetes or Firmicutes was the TBI-0 group. While some ASVs are characteristic of multiple treatment groups (e.g., Family Muribaculaceae demarcates the TBI-90, Con-45 and Con-90 groups), these ASVs are nevertheless different. Despite the fact that very few significant differences were seen in the rmTBI and control groups at any time point, the LEfSe analysis shows that each treatment group is characterized by a specific set of taxa.

All ASV DNA sequences in Fig. 5 above were submitted to the NCBI basic local alignment search tool (BLAST) for taxonomic identification and 3 ASV sequences matched with 99.6 to 100% identity, with the species *Muscispirillum schaedleri* (ASV 003) for the TBI-0 group, and with *Bacteroides ovatus* (ASV 078) and *Flintibacter butyricus* (ASV 188) for the Con-0 group. Many other potential matches were uncovered but the sequence alignments were less than 99% so species identification could not be made reliably.

#### Phylotype analysis of the gut microbiota in rmTBI

**Figure 6:**
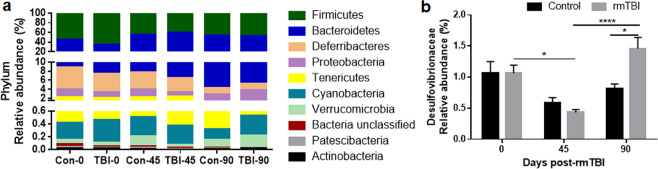
Percent relative abundances of phyla in the treatment groups. Stacked columns for the 10 most prominent bacterial phyla are included (**a**). The relative abundance of taxa below the level of phylum (**b**) are presented as mean % ± SEM. The symbols indicate the levels of significance as follows for the indicated comparisons: ****p < 0.0001 and *p < 0.05.

Figure 6a shows treatment effects at the phylum level and indicates that statistically significant changes in relative abundance were restricted to the Bacteroidetes and Firmicutes phyla. Two-way ANOVA analysis indicated that the main effect of treatment was not significant but the main effect of phylum (F(9,290) = 917, p < 0.0001) and the phylum X treatment interaction (F(45,290) = 8.14, p < 0.0001) were significant. *Post hoc* analyses showed that rmTBI differed significantly from its control only at the 0-day time point for both Firmicutes (p < 0.001; Tukey’s test) and Bacteroidetes (p < 0.01; Tukey’s test). Otherwise, the analyses of phylotypes below phylum agreed well with those shown above. For instance, each rmTBI group differed from the other rmTBI groups but not from the respective time-paired control group. Likewise, all control group comparisons were significantly different from each other for both phyla with the exception of the Con-45 vs the Con-90 groups. The results of all pairwise *post hoc* comparisons for % relative abundance of Bacteroidetes and Firmicutes phyla are presented in Supplemental Table 1. Figure 6b presents the differences on the relative abundance of members from the Desulfovibrionaceae family. There was a significant main effect of time (F(2,29) = 13.74, p < 0.0001), and a treatment X time interaction (F(2,29) = 5.04, p < 0.05), whereas the main effects of treatment did not reach significance (Fig. 6b). Pairwise *post hoc* comparisons showed statistical differences between the control and rmTBI groups at 90 days (p < 0.05). Additional significant comparisons in Desulfovibrionaceae included TBI-0 and TBI-45 (p < 0.05), and TBI-45 and TBI-90 (p < 0.0001). Other selected taxa below the phylum level that showed statistical differences are presented in Supplemental Fig. 2. Neither the main effects of treatment nor the treatment X time interaction was significant for the relative abundance of the classes Bacteroidia and Clostridia. Nonetheless, the effects of time were significant for both classes (F(2,29) = 17.43, p < 0.0001 for Bacteroidia, F(2,29) = 16.83, p < 0.0001 for Clostridia).

## Discussion

The goals of the present study were to 1) determine if repeated head impacts with a low mass weight would impart a very mild brain injury that resembled pathologies as seen in humans and 2) determine if rmTBI could lead to alterations in the gut microbiota. The rationale for the present studies is based on the recognition that alterations in the gut microbiota are associated with numerous neurodegenerative (e.g., Parkinson’s disease, Alzheimer’s disease) and neurodevelopmental disorders (e.g., autism) as well as with stroke and brain injury^40^. With regard to the first goal, the results indicated clearly that a total of 20 head impacts caused slowly evolving neuropathological changes. During the course of head impacts, the initial and large increase in ROR eventually returned to control levels after 12 impacts, suggesting an adaptive or tolerance-like response such that mice exposed to rmTBI were not different from controls nearing the end of the course of treatment. This response is not preconditioning (i.e., treatment with a small dose of an otherwise harmful stimulus that causes tolerance to a subsequent injurious event), as has been used in studies of ischemic stroke^41^ because we used the same head impact conditions (30 g drop weight) throughout treatment. Some of the behavioral symptoms associated with CTE include cognitive impairment^42^, and evidence from preclinical reports of rmTBI outcomes consistently include cognitive deterioration^43,44^. Thus, we evaluated the effects of rmTBI on recognition memory and found injury-related alterations at the 45- and 90-day time points. This is indicative of a functional impairment that required time to develop as the group tested shortly after head injury was not affected. Brain examination for signs of white matter pathology indicated that microglial activation was evident quickly after the last head injury and persisted until the late 90-day point. Astrocyte reactivity was observed at 0 days after rmTBI but did not become significant until 45 and 90 days after the last head impact. While p-tau expression in the optic tract increased at 45 days post-injury, it became significantly evident at the 90-day time point. While increases in p-tau have been documented with other mouse models of rmTBI with^43^ and without white matter damage^44^, these alterations were reported for gray matter. It is not clear whether these increases in hyperphosphorylated protein were circumscribed to gray matter only or whether a white matter evaluation was spared. In our model, all the observed alterations are indicative of a progressive neuropathology that starts soon after rmTBI but intensifies over time. These slowly evolving neuropathological signs resemble what has been reported in military personnel and athletes exposed to rmTBI throughout their careers^45–49^. We attribute this progressive emergence of white matter neuropathology to the rmTBI method used presently. The key characteristics that make this model ideally suited for studying military- and sports-related rmTBI, and that distinguish it from existing animal models^50^, include infliction of a very mild injury, use of unrestrained mice to allow rotational acceleration of the head upon impact, and the use of numerous head impacts. We observe extensive white matter reactive gliosis and increases in p-tau and TDP-43^31,32^, in keeping with changes seen in blast-related TBI in deployed military personnel^47,51^ and athletes participating in collision sports^45^. White matter inclusions are appreciated for their importance in determining clinical outcome in terms of cognitive and functional impairment^45^ and it is now known that white matter abnormalities can worsen over long periods of time after TBI^52^.

With regard to the second goal, and based on emerging literature showing acute and chronic gut microbiota dysbiosis after TBI in humans^30^ and rodents^26–29^, it was hypothesized that rmTBI would also cause significant alterations in the gut microbiota that would parallel the emergence of neuropathological alterations. The results did reveal minor alterations in community diversity (α- and β-) that were influenced more by time than by treatment. In fact, the only time at which rmTBI differed from controls was the 0-day time point when significant alterations in the Bacteriodetes and Firmicutes phyla emerged. Although reports on the effects of age on the gut microbiome of young mice are scarce, a study in the C56BL/6 strain indicates that the relative abundances of bacterial populations vary from an early post-weaning period throughout middle age^53^. These bacterial communities were relatively more stable in a late-period group (141–150 days post-weaning -dpw-) compared to an early-period group (0–9 dpw), but differences were still present at the taxonomical level. The subjects age in the present study ranged from around 63 dpw (0 days), 108 dpw (45 days) and 153 dpw (90 days), and while no specific age comparisons were possible with the above-mentioned study due to their wide age arrays, it could still be inferred that age-driven bacterial community variation could have contributed to the differences in gut microbiota we observed at each time point. When levels below phylum were examined, the only instance when time-paired rmTBI and controls differed significantly was the 90-day time point, showing that the family Desulfovibrionaceae (phylum Proteobacteria) was significantly increased in percent relative abundance in the rmTBI group versus its control. Studies on the specific effects of the Desulfovibrionaceae family increases are scarce but it was reported that transgenic mice with cognitive impairment used as models of Alzheimer’s disease had an increased abundance in this particular bacteria group^54^. While we observed recognition memory deficits induced by rmTBI at the same 90-day time point, it is unclear whether the increases in Desufovibrionacea are directly related to cognitive deficits given that these bacteria were not altered at the 45-day time point, when memory was also impaired. Several factors could account for the relatively immediate effect of rmTBI on the gut microbiota. First, previous studies showing gut microbiota dysbiosis in animals after TBI used single head impacts that imparted severe injuries and assessed outcomes after relatively short times after TBI (1–7 days^26–29^. In fact, Nicholson and colleagues^29^ noted that changes in the gut microbiota in their studies were significantly positively correlated with lesion volume. Humans with chronic dysbiosis had experienced severe TBI as well^30^. The one study that exposed rats to repeated heat impacts (N = 5) used our rmTBI method^31–33^ and reported alterations in microbial diversity out to 30 days after the last head impact^55^. Unfortunately, this study did not include data showing that the head impacts caused brain damage so it is difficult to judge the extent to which changes in the gut microbiota can be attributed to TBI^55^.

A second possible reason we did not presently see alterations in the gut microbiota of the same magnitude reported in previous studies could relate to diet and nutrition. It is known that moderate to severe TBI and other hypercatabolic conditions are characterized by anorexia, body weight loss and muscle protein breakdown^56^, alterations that are difficult to reverse with standard enteral diets. This condition has been referred to as “resistance to renutrition”^20^ and reflects the increased emphasis on using enhanced nutritional intervention as part of the therapy for TBI^57,58^. In light of the profound influence diet and nutrition can have in reshaping the gut microbiota^59^, it is possible that prior preclinical and clinical studies of gut microbiota disruptions after a single, severe TBI could reflect indirect effects of nutritional alterations on the microbial community and not a direct effect of head injury. Of the preclinical studies referenced above^26–29,55^, Matharu and colleagues were the only ones to report body weight changes after TBI, but the reductions noted in this study were non-significant. Therefore, it cannot yet be ruled out that TBI-induced alterations in feeding or nutritional status are causing the changes in the gut microbiota that accompanies TBI. Although we did not measure body weight in the current study, results from our previous work using a more drastic rmTBI paradigm (30 head impacts with a 95 g weight) did not show differences in subjects exposed to rmTBI compared to controls (mean ± SEM were the following: 23.7 g ± 0.397 for controls, 24 g ± 0.437 for rmTBI mice). Thus, we did not expect a milder paradigm to cause any body weight differences. All this highlights the need for studies that evaluate the effects of very mild and repeated TBI on the intestinal microbiota. To our knowledge, the present study is one of the few that describe such effects in the gut and intended to draw possible associations with the neuropathological and behavioral outcomes of the injury.

## Conclusion

The results from the present studies lead to the conclusion that rmTBI causes recognition memory impairments at late time points as well as progressive damage in the optic tract. This impairment process initiates with microglial activation at the 0-day time point and persists until 90 days. In addition, increases in astrocyte reactivity are evident from the intermediate time point post-injury until the late point. These changes culminate with substantial increases in p-tau expression at 90 days. Despite these CNS changes, rmTBI does not cause alterations in the gut microbiota of the magnitude seen after single, severe head injuries. While we did observe a limited number of rmTBI-induced alterations in the diversity and structure of the microbial community, the most apparent changes seen in subjects were related to time passage and not rmTBI. The fact that most changes in the gut microbiota were time-related was not surprising in light of evidence indicating that the host gut microbiota evolves during aging^60,61^. Therefore, it appears from our studies that the CNS alterations and changes in the gut microbiota were varying independently. Additional studies will be required to determine if TBI-induced alterations in the gut microbiota are direct or are mediated indirectly via changes in nutrition.

## Supplementary information

Supplementary information.
