# Supplementary material for: Repetitive, mild traumatic brain injury results in a progressive white matter pathology, cognitive deterioration, and a transient gut microbiota dysbiosis
Source: Sci Rep. 2020 Jun 2;10:8949. doi: 10.1038/s41598-020-65972-4 (PMC7265445; doi:10.1038/s41598-020-65972-4)
Supplement: Supplementary file 1 — Supplementary information. [file 41598_2020_65972_MOESM1_ESM.docx]

Repetitive, mild traumatic brain injury results in a progressive white matter pathology, cognitive deterioration, and a transient gut microbiota dysbiosis

Mariana Angoa-Pérez, Branislava Zagorac, John H. Anneken, Denise I. Briggs, Andrew D. Winters, Jonathan M. Greenberg, Madison Ahmad, Kevin R. Theis, and Donald M. Kuhn

Fig. S1. Heat map illustrating patterns in ASV relative abundance among the treatment groups. All subjects in each group are arrayed in columns and bacterial taxonomies are indicated in rows. Clustering along the y-axis was done using the Ward algorithm. Groups are indicated as follows: Con-0 (control-0 days), TBI-0 (rmTBI-0 days), Con-45 (control-45 day time point), TBI-45 (rmTBI-45 day time point), Con-90 (control-90 day time point) and TBI-90 (rmTBI-90 day time point).


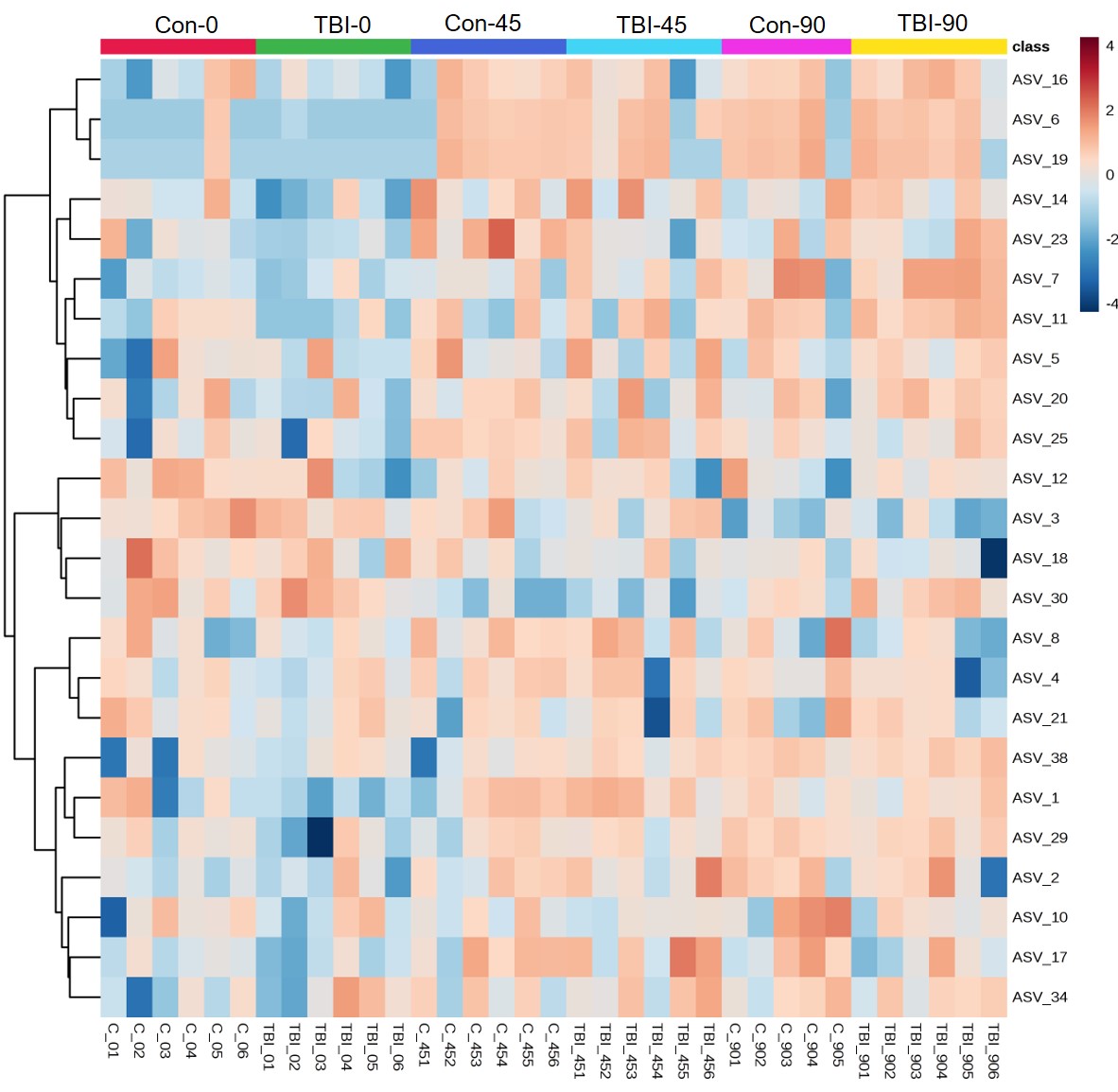


**Fig. S2.** Relative abundance of taxa below the level of phylum in treatment groups. Results are presented as relative abundance for each taxon. Groups are indicated as follows: Con-0 (control-0 days), TBI-0 (rmTBI-0 days), Con-45 (control-45 day time point), TBI-45 (rmTBI-45 day time point), Con-90 (control-90 day time point) and TBI-90 (rmTBI-90 day time point). The symbols indicate the levels of significance as follows for the indicated comparisons: **p<0.01 and ***p<0.001.


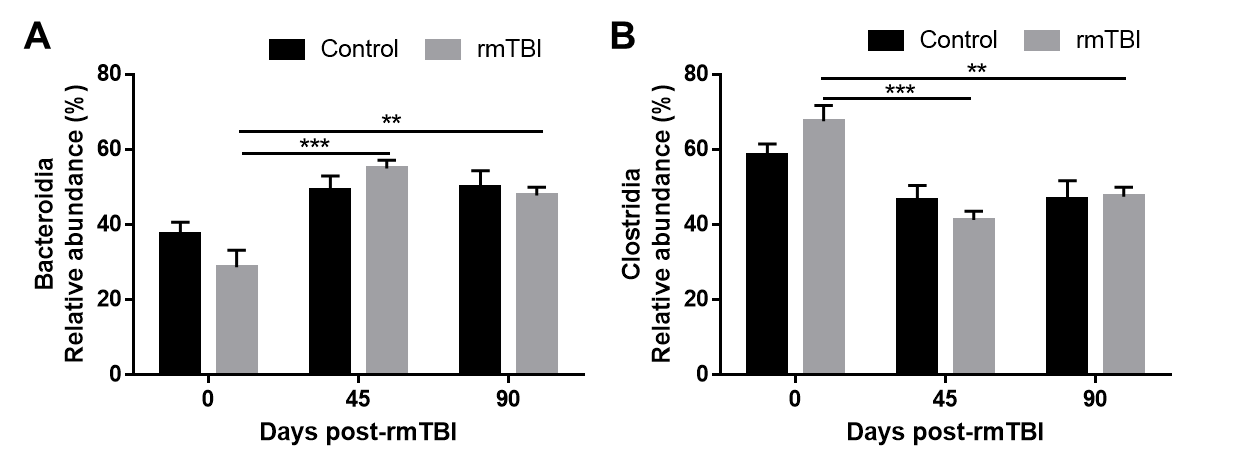


**
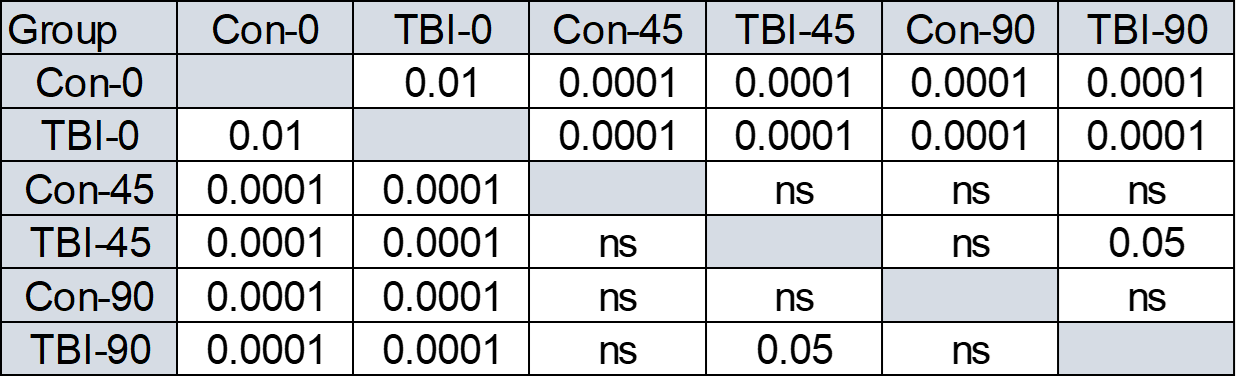
**

Table S1. p values for pairwise comparisons (two-way ANOVA) among groups at the level of bacterial phyla. Not significant values are represented as ns. Groups are indicated as follows: Con-0 (control-0 days), TBI-0 (rmTBI-0 days), Con-45 (control-45 days), TBI-45 (rmTBI-45 day time point), Con-90 (control-90 day time point) and TBI-90 (rmTBI-90 day time point).
